# Determination of the Personal Nutritional Status of Elderly Populations Based on Basic Foodomics Elements

**DOI:** 10.3390/foods10102391

**Published:** 2021-10-09

**Authors:** Natalija Uršulin-Trstenjak, Ivana Dodlek Šarkanj, Melita Sajko, David Vitez, Ivana Živoder

**Affiliations:** 1 Department of Food Technology, University Center Koprivnica, University North, Trg dr. Žarka Dolinara 1, 48000 Koprivnica, Croatia; natalija.ursulin-trstenjak@unin.hr; 2 Department of Nursing, University Center Varaždin, University North, Jurja Križanića 31b, 42000 Varaždin, Croatia; msajko@unin.hr (M.S.); izivoder@unin.hr (I.Ž.); 3 County Hospital Čakovec, I.G.Kovačića 1e, 40000 Čakovec, Croatia; david.vitez.7@gmail.com

**Keywords:** elderly population, individual nutritional status assessment, elderly homes, exposure, foodomics

## Abstract

Nutritional status is a series of related parameters collected using different available methods. In order to determine the nutritional status of elderly populations and ensure nutritional support based on an individual approach, the implementation of the increasingly used foodomics approach is available; this approach plays a key role in personalized diets and in the optimization of diets for a population group, such as an elderly population. The Mini Nutritional Assessment (MNA) method and the Nottingham Screening Tool (NST) form were used on 50 users in a home for the elderly in northwest Croatia. A loss of body mass (BM) was statistically significantly higher for those who had the following: decreased food intake in the last week and users who had complete and partial feeding autonomy. Additionally, the obtained data on drug intake, fluid, individual nutrients, and physical activity are based on an individual approach. The available documentation provides insight into nutritional values and food preparation in an attempt to satisfy a holistic approach in the evaluation of exposure while trying to achieve as many elements of foodomics as possible.

## 1. Introduction

People over 65 years of age require a routine nutritional status assessment at least once a year, while those older than 75 years require more frequent assessments [1]. Numerous indicators are used in such assessments, such as anthropometric measurements, laboratory parameters, clinical examination, function tests, and questionnaires, all of which provide a guaranteed approach to each user/respondent. Foodomics plays a crucial role in personalized diets and the optimization of diets for groups of the population, an example of which is the elderly. The increasing frequency of impaired nutritional status has prompted the innovation of simple and effective patterns as a screening to detect both malnutrition and obesity among the general, hospitalized, and institutionalized older population. These screenings contain questions about inadvertent weight loss (further body mass—BM) and body mass index (BMI), dietary habits, and functional status. They represent an insight into the need for either the introduction of nutritional support or the use of an appropriately prescribed diet [2,3].

The four questions in each pattern relate to a loss of BM over the last three months, food intake, BMI, and disease progression. The British Society for Parenteral and Enteral Nutrition and its experts put these issues together and use them in the Nottingham Screening Tool (NST). New and more comprehensive nutritional assessment forms applicable in hospitals, elderly homes, and the community have emerged from the following: (1) Subjective Global Assessment (SGA), which is a fast, widely available, and inexpensive technique with good reproducibility; (2) a validated method, namely the Mini Nutritional Assessment (MNA); (3) a universal form, i.e., the Malnutrition Universal Screening Tool (MUST) containing a five-step assessment algorithm; and (4) a nutritional risk assessment tool—Nutritional Risk Screening (NRS 2002), which was developed and validated as a questionnaire by the European Society for Clinical Nutrition and Metabolism (ESPEN) [3,4].

Impaired nutritional status adversely affects physical and mental health, resulting in increased complications, higher mortality rates, and increased treatment costs. Malnutrition patients are more susceptible to infection and other health-related complications. In healthy older individuals, malnutrition decreases quality of life and increases the risk of sarcopenia and bone fragility [5], while obese people are exposed to cardiovascular disease, diabetes, and renal dysfunction [6,7,8,9]. An essential problem in assessing nutrient intake its interaction with antinutrients [10], heavy metals [11], mycotoxins [12,13,14,15], and pesticides [16] that, depending on their size [17,18,19], can be found in macroscopic clusters or nanoparticles. Thus, a more holistic approach for assessing exposures has recently been advocated, one that estimates intakes and interactions between particular food components [20]. All of these phenomena, together with inherent properties assessed by whole-genome sequencing (WGS) or single-nucleotide polymorphism analysis (SNP), can help in creating personalized nutrition plans that, combined with foodomics, can, in theory, provide the best results for preserving the health of older populations.

Considering that users in elderly homes are a population with long-standing habits of consuming a particular, traditional diet, it is essential to balance and meet the needs of these users through technological preparation and the processing of food, respecting the energy and nutritional value of a given meal [21]. Considering that this is a sensitive population, much attention should be paid to the implementation of a hazard analysis and a critical control points (HACCP) system to ensure the health and safety of the technological preparation of a meal while respecting ethical manufacturing and hygiene practices [22,23]. Additionally, the highest priority should be the respect of users’ wishes and preferences, which are emphasized in the ESPEN guideline; this is particularly important in homes for elderly individuals [4].

Foodomics encompasses analytical platforms for researching food composition and, with this, its nutritional values and impact on health are demonstrable. New techniques also provide insight into the detailed picture of food quality and can be used to discover food deceits, as well as to find solutions for other challenges that arise in food production. Foodomics was introduced at an international conference in 2009 in Cesena, Italy, and it was defined as “a discipline that studies areas of food and diet by using and integrating advanced –omics technologies with the purpose of advancing the well-being, health and knowledge of consumers”. It requires a combination of food chemistry, biological sciences, and analysis of dates. It encompasses the four main areas of omics: genomics, transcriptomics, proteomics, and metabolomics [24]. Generally, the results of Foodomics’s research have a direct impact on consumers, the food industry, and society at large [24]. Regardless of the fact that foodomics is a relatively new research discipline, it is developing fast and expectations related to its growth are high. It is believed that it will become an effective tool for the development of healthy food that is adapted to individual health challenges, and that it can help in preventing illnesses connected to food and food deceits [25,26].

## 2. Materials and Methods

This research aims to apply a form for assessing nutritional status, eating habits, and health status of users/respondents (after this referred to as users) in elderly homes. We seek to obtain insights not only into the nutritional value of prepared meals, but also regarding the intake and interaction between individual portions of food by assessing exposures, as well as the application of the HACCP system, which ensures the correct preparation of meals in these institutions. The survey included 50 users in elderly homes in the Republic of Croatia, and it was conducted between October and December 2019 with the consent of their institutions.

Through the research, which was conducted between October and December 2019, 50 users of nursing homes in the Republic of Croatia have been surveyed with the approval of the institution. This research was conducted using tools and forms used for evaluating nutritive status (in hospitals, nursing homes, and in the care for elderly people in the community). Four questions were considered in each of the forms, and they were related to a loss of BM in the last three months, BMI, food consumption, and the advancement of illness. According to experts from the British Association for Parenteral and Enteral Nutrition, those questions are connected in the Nottingham Screening Tool-NST. As well as these data, the questionnaire (Questionnaire S1) also included general data about the examinee: sex, age, body weight, body height, diagnosis, and ITM [2]. New and more extensive forms for the evaluation of nutritive status have been developed out of this form and these were also used in this research. Those forms are the following:

Subjective Global Assessment—SGA—is a widely used and cheap technic that connects data from a clinical examination and the anamnesis of an illness, through which a quick assessment of nutritive status can be obtained and, if needed, a quick intervention can be provided. Insight is obtained regarding a loss of BM, a change in food consumption, relevant gastrointestinal symptoms, functional status, and metabolic needs of a patient. Occurrence of infection, length of hospital stay, serum albumin and transferrin, muscle strength, and other objective parameters of the SGA method all influence the obtained data [2].

Mini Nutritional Assessment—MNA—is a quick method used with elderly people in hospitals and nursing homes, as well as with people living alone. The goal of this method is the evaluation of the risk of malnutrition and the insurance of early nutritive support. It encompasses anthropometric measurements (BM, body height (BH), scope of the upper arm), short questions for general evaluation (7 questions concerning a loss of body mass, lifestyle, drug intake, and mobility), questions about nutritional intake (8 questions concerning number of meals, food and water intake, and the possibility of autonomous feeding), and a patient’s self-assessment of their nutritive and health status [1]. The obtained data are different depending on the conditions under which the MNA is conducted; therefore, this tool is considered to be at its most accurate when conducted with people who live independently from a broader community because of examinee cooperation [2].

Malnutrition Universal Screening Tool—MUST, although it is primarily intended to be used within the elderly population, also has its uses in whole hospital populations. It is comprised of 5 steps, and uses ITM, loss of body mass, and the impact of the degree of the illness on the health of a patient for determining nutritive status [2].

The tool, that is, the Nutritional Risk Screening questionnaire, or NRS, was developed in 2002, and it is comprised of two parts and is recommended by the ESPEN [4]. The first part is comprised of four questions: (1) Is the patient’s BMI lower than 20.5? (2) Has the patient inadvertently lost body mass in the last four months? (3) Did the patient have a small food intake in the last week? (4) Is the patient gravely ill? (for example, intensive care, chronic disease) [2].

In case of an affirmative answer to any of those questions, the examiner continues with the second part of the questionnaire that takes a closer look at the evaluation of nutritive status. Once all data are obtained, a classification is established as to whether the patient is exposed to nutritive risk or whether their nutritive status should be monitored once a week [1].

Through such a data collection method, an individual and comprehensive approach is emphasized in developing diets for elderly users in nursing homes; this approach has been applied in this paper.

For nutritional status assessment, we used the Nottingham Screening Tool (NST) questionnaire containing user information (gender, age, body mass (BM), height, diagnosis), body mass index (BMI), data on accidental loss of BM and food intake, and disease severity data, and a rapidly validated method for assessing nutritional status (Mini Nutritional Assessment, MNA). These tools were used based on anthropometric measurements (BM and body height (BH), upper arm circumference), general assessments (7 questions related to BM loss, lifestyle, medication, and mobility), dietary intake questions (8 questions regarding meal count, food and water intake, and self-feeding options), and patient self-assessment (how the patient perceives his or her nutritional and health status) [1]. Such a way of collecting data is attuned to an individual and comprehensive approach in the creation of a diet for users in elderly home.

Based on the above, an individual questionnaire of 24 questions was created. The first two questions relate to the gender and age of the respondents and the other two to their anthropometric measurements (BM and BH). The following are a series of grouped questions. The first group includes questions about the loss of BM, the number and cause of weight loss, taking medication and lifestyle, and physical activity. The second group includes questions about the dietary intake of the users, namely the number of meals and fluid intake, and the possibility of self-feeding. The third group of questions is about the duration of and recent changes in diet. In a separate question, users’ self-assessment of their health status, gastrointestinal symptoms, and metabolic needs were examined in relation to illness. The research described in this paper shows a correlation between patients’ health status and their nutritional status (Figure S1).

The following software packages were used for statistical data processing and graph creation: Microsoft Excel 2016 (Microsoft, Redmond, WA, USA), Statistica 13.3 (TIBCO Software Inc., Palo Alto, CA, USA), Tableau Desktop 2020.4 (Tableau software, Seattle, WA, USA), and Flourish studio. For statistical tests, the normality of data distribution was examined with Shapiro–Wilks’ W test; a Leven test confirmed the homogeneity of variance. Given the results of these tests, the Student’s t-test or the Mann–Whitney U test was used for a comparison of the two groups, and comparisons of multiple groups were conducted with ANOVA and Kruskal–Wallis ANOVA, respectively. The results were considered statistically significantly different when *p* ≤ 0.05.

## 3. Results

### 3.1. Distribution of Users According to BMI

**Figure 1:**
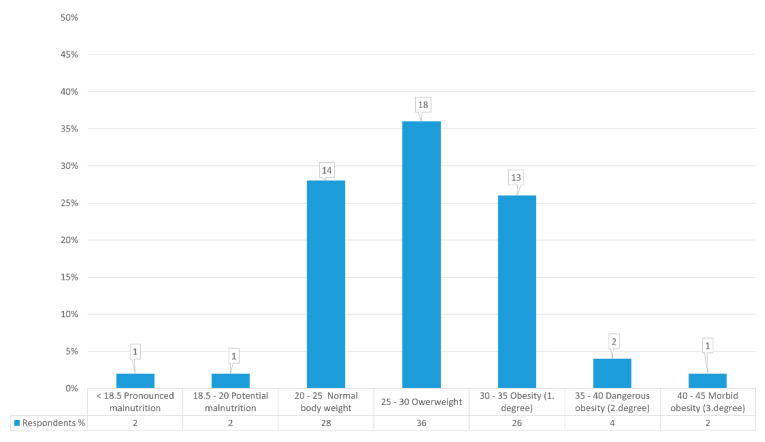
Distribution of users according to body mass index (BMI) shown in absolute and relative terms.

The BMI calculation formula provides an index for all 50 survey users categorized as nutritional categories. The data obtained show a statistically normal distribution (Figure 1).

### 3.2. Loss of BM in Consumer Group Considering Food Intake and Users in Terms of the Possibility of Consuming a Meal

**Figure 2:**
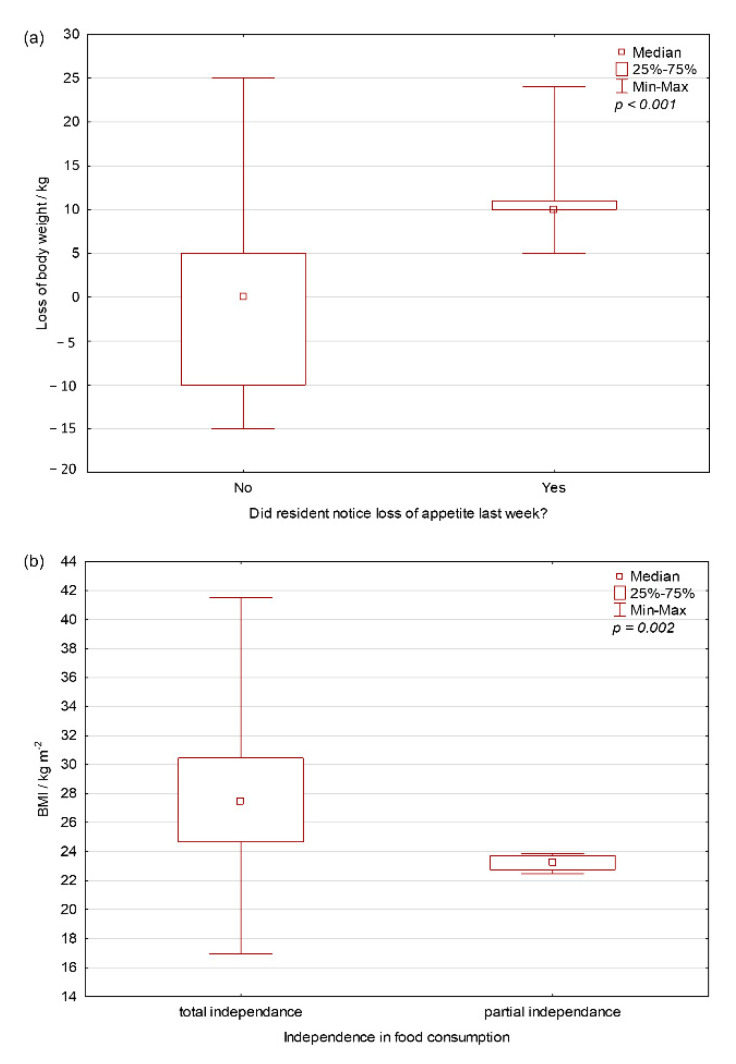
Loss of body mass: (**a**) comparison of user groups concerning food intake; and (**b**) comparison of users about the possibility of consuming meals. The groups were compared by Mann–Whitney U test and shown to be statistically different (*p* values are on graphs).

The loss of BM within a one-month period was statistically significantly higher in those with reduced food intake in the previous week (Mann–Whitney U = 43.5; *p* < 0.001) (Figure 2). There was also a statistically significant difference in the distribution of data between users with complete autonomy and users with partial autonomy in feeding (*p* = 0.002).

### 3.3. Comparison of the Number of Daily Meals and BM, Common BM, BH, and BMI

**Figure 3:**
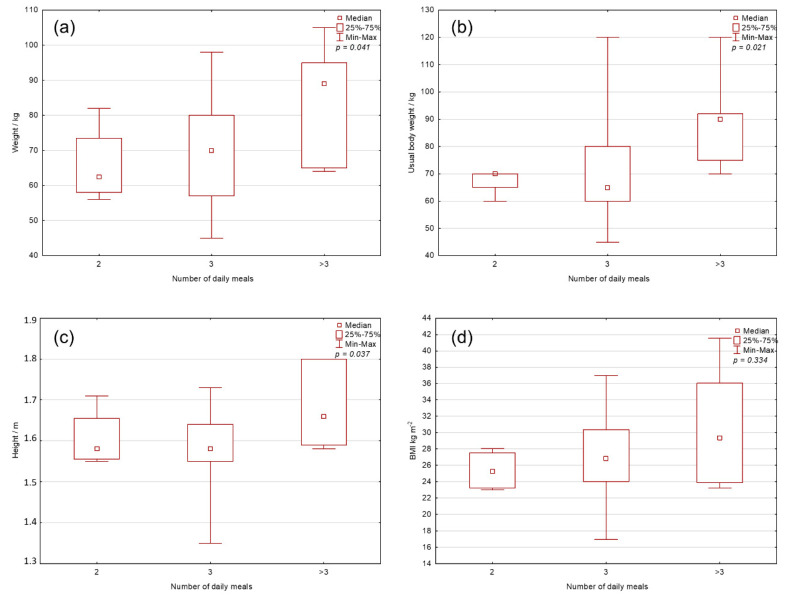
Comparison of the number of daily meals and: (**a**) body mass; (**b**) common body mass; (**c**) height; and (**d**) body mass index. The groups were compared by Mann–Whitney U test (*p* values are on graphs).

Comparing the number of meals that users consume (Figure 3) shows that there is a statistically significant difference between the number of daily meals and BM (*p* = 0.041), the usual BM (*p* = 0.021), and BH (*p* = 0.037), but interestingly not with BMI (*p* = 0.334). BH is well correlated (r = 0.5604; *p* < 0.001) with BM, which is why there is no significant difference in BMI.

### 3.4. Distribution of Users According to BMI Groups and Gastrointestinal Symptoms

**Figure 4:**
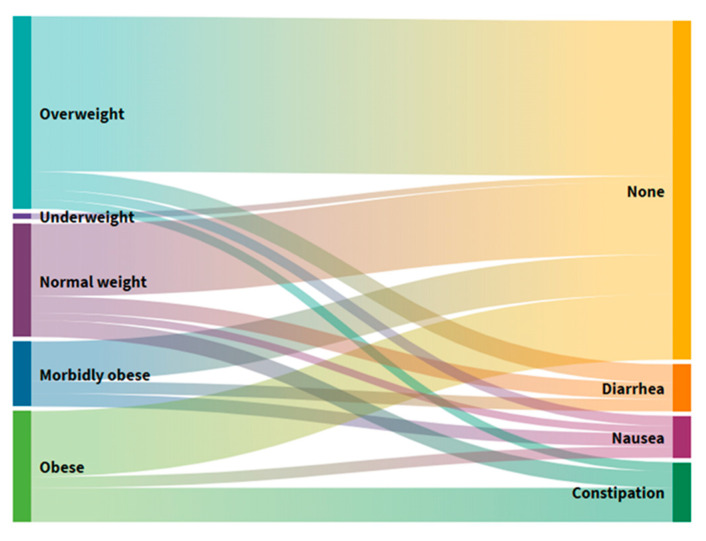
Alluvial diagram of the distribution of users according to the BMI groups and gastrointestinal symptoms.

Comparing users divided into BMI groups (Figure 4), 69.5% of participants do not have any gastrointestinal symptom problems in each BMI group, while constipation is the most frequently reported gastrointestinal symptom in the group characterized by dangerous obesity; nausea in the morbid obesity group; and diarrhea in the normal and excessive BM group.

### 3.5. Distribution of Users by the Proportion of BMI Groups and Self-Reported Cause of Weight Loss

**Figure 5:**
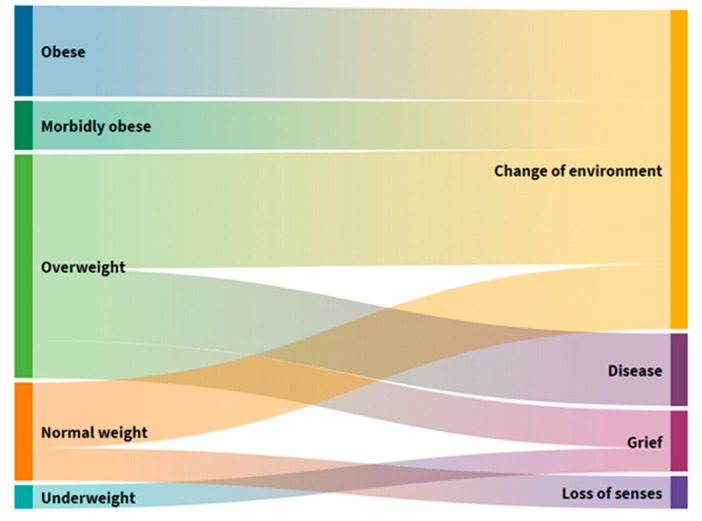
Alluvial diagram of the distribution of users by individual BMI groups and self-reported weight loss cause.

Figure 5 shows the distribution of the stated mass loss concerning self-assessment. 

### 3.6. Distribution of Users According to Individual BMI Groups and Consumption of More Than Three Different Medicines

**Figure 6:**
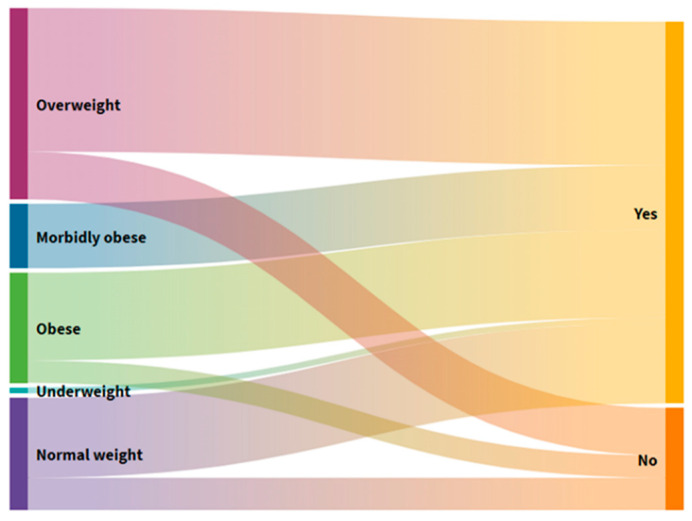
Alluvial diagram of the distribution of users by individual BMI groups and consumption of more than three different drugs.

Users (Figure 6) in the morbidly obese group consumed more than three drugs, the most among all groups. Fewer than three drugs were taken in the group with normal and excessive BM.

### 3.7. Distribution of Users by Individual Groups and Fluid Intake

**Figure 7:**
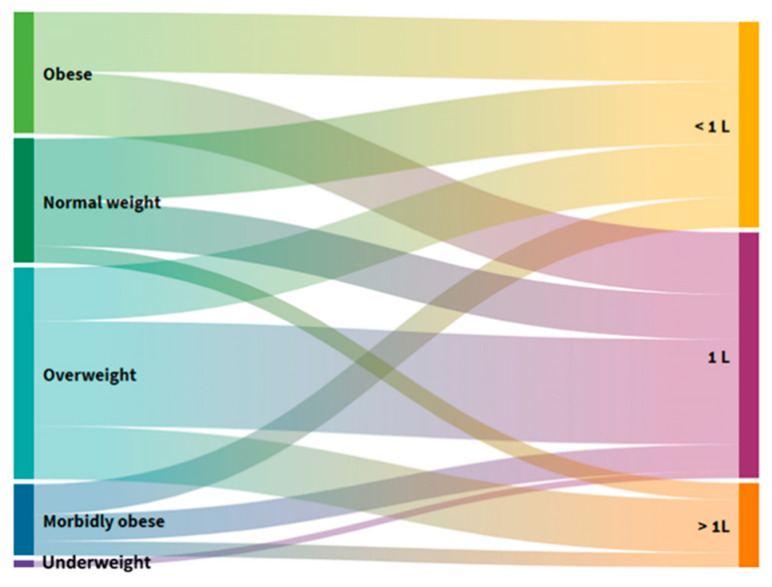
Alluvial diagram of the distribution of users by BMI groups and fluid intake.

Figure 7 shows that users with reduced and normal BM take less than 1 L, and those with elevated and excessive BM more than 1 L.

### 3.8. Distribution of Users According to the Frequency of Intake of Macronutrients, Milk and Milk Products, Vegetables, and Fruits Absolutely and Relatively

**Figure 8:**
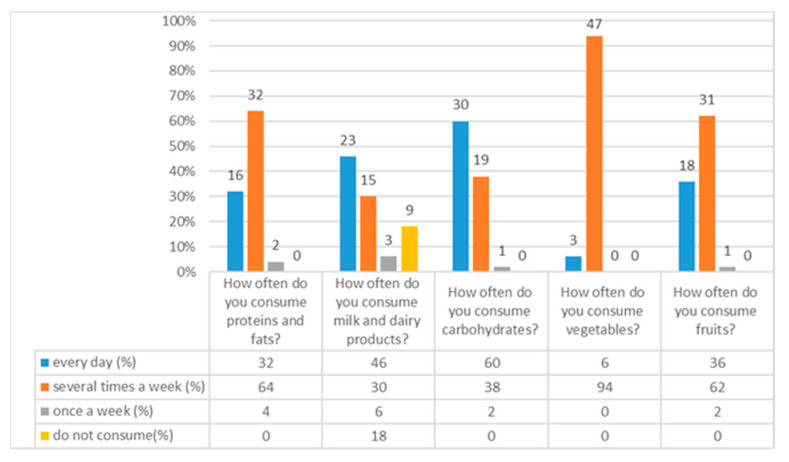
Distribution of users by frequency of intake of macronutrients, milk, and milk products, vegetables, and fruits absolutely and relatively.

In the Figure 8 distribution of user’s macronutrients, milk, and milk products, vegetables, and fruits are shown. Major proportion are consuming milk and milk products, and carbohydrates every day, while proteins and fats, vegetables and fruits are consumed mainly several time a week.

### 3.9. Comparison of the Reduction in Intake of Each Food Group with the Loss of BM Concerning BMI Group

**Figure 9:**
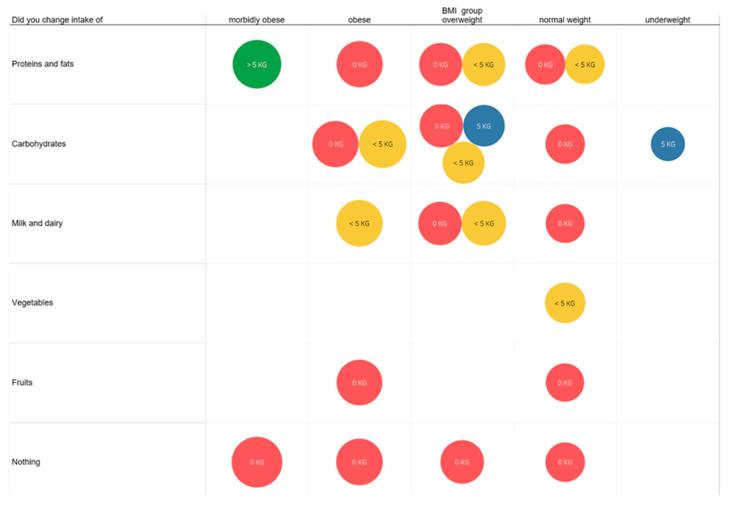
Comparison of dietary intake reduction with weight loss concerning BMI group. The color of bubbles is coded according to weight loss (red—0 kg loss; yellow—up to 5 kg loss; blue—5 kg loss; green—more than 5 kg loss).

The labels on the Figure 9. show the following: >5 kg—users lost more than 5 kg; 5 kg—users lost 5 kg; <5 kg—users lost less than 5 kg; 0 kg—users did not lose weight. Users also reported changes in their diet and the elimination of or reduction in consumption of a particular group of foods.

### 3.10. Distribution of Users by BMI Groups by Frequency of Intake of Macronutrient, Milk and Dairy Products, Vegetables, and Fruits

**Figure 10:**
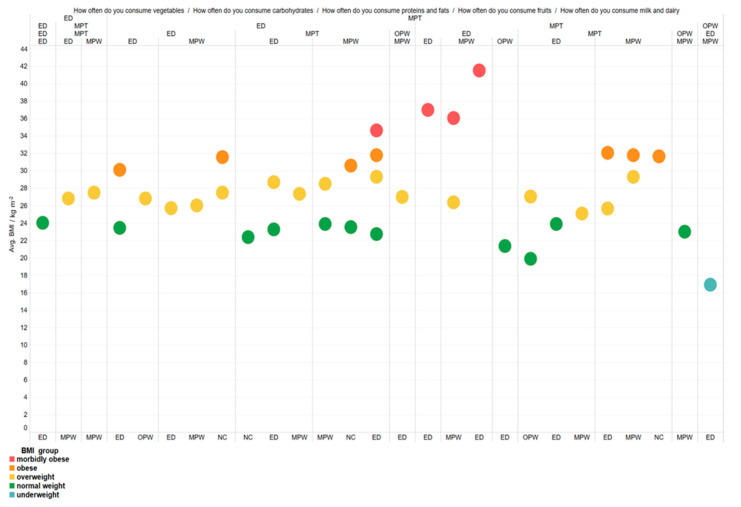
Distribution of users by BMI groups by frequency of intake of macronutrient, milk and dairy products, vegetables, and fruits.

The Figure 10. is divided down by the frequency of food entries from the heading of the graph, using the following abbreviations: ED—every day; MPT—multiple times per week; OPW—once per week; NC—not consuming.

### 3.11. Distribution of Users according to BMI Groups and Physical Activity (Alone or with Help)

**Figure 11:**
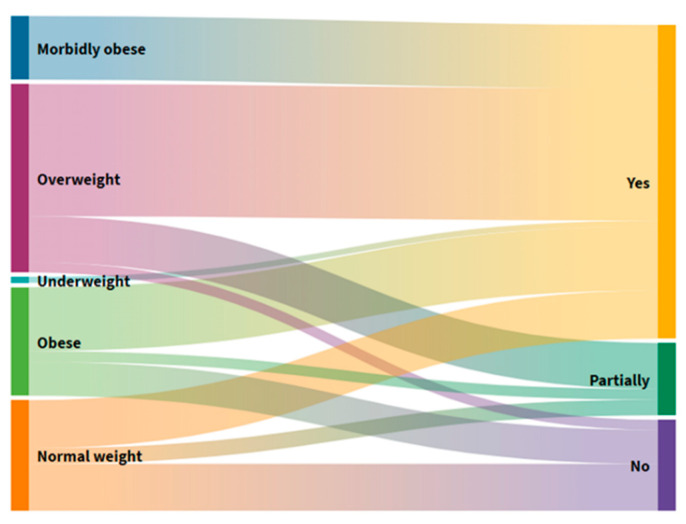
Alluvial diagram of the distribution of users according to the BMI groups and physical activity (alone or with the help).

Everyone with morbid obesity (Figure 11), dangerous obesity, and excessive BM engaged in some physical activity, while users with normal BM avoided sports to the fullest.

## 4. Discussion

This study shows that, out of all the users, three (6%) confirmed that therapy affected BM loss, going on to list common gastrointestinal symptoms such as nausea and diarrhea, while 47 (94%) stated that treatment did not affect their weight loss (Figure S2). More than 250 medicines can affect the absorption and excretion of nutrients. Other side-effects of drugs adversely affecting nutritional status are anorexia (acetylcholinesterase inhibitors, antibiotics, digoxin, hypnotics), early satiety (anticholinergics, sympathomimetics), reduced feeding ability (sedatives, opiates), dysphagia (Nonsteroidal anti-inflammatory drugs (NSAIDs)), constipation (diuretics), and diarrhea (laxatives, antibiotics) [27]. This research was conducted with 50 respondents/users from an elderly home and included 10 (20%) men and 40 (80%) women. According to BMI values and nutritional status, they are classified into nutritional categories with absolute and relative representation. Of the total, only one user was highly malnourished, with a BMI < 18.5 kg/m^2^, or 16.94 kg/m^2^. One user with a BMI < 20 kg/m^2^ and 19.88 kg/m^2^ also had potential malnutrition. There were 14 users with normal BMI, (28%) from 20 kg/m^2^ to 25 kg/m^2^. Eighteen users (36%) were overweight, exceeding the BMI limits of 25 kg/m^2^ to 30 kg/m^2^. That means that 13 (26%) users are in Grade I obesity and 2 (4%) are in Grade II. One user is in Grade III, which signifies morbid obesity with a BMI > 40 kg/m^2^ (Figure 1). In Croatia, the situation is better when compared to a Korean study from 2016 that observed malnutrition in 31% of its examinees, while 49% were at risk of malnutrition and 16% had a normal nutritive status [28]. Additionally, results obtained in Germany from 188 examinees in two elderly homes confirmed that 57.4% were at risk of malnutrition and 15.4%, were malnourished; only 27.1% had a normal nutritive status [29]. The difference in obtained values can be explained by considering that the number of examinees in the mentioned and compared examples was higher. It can also be explained with the use of different tools for the evaluation of the nutritive status within elderly populations and the different degrees of care in different research undertakings [30].

Research has shown that appetite declines with age, as demonstrated by a comprehensive survey by Giezenaar et al. [30]. Although the loss of BM over the observation period provides essential information about the nutritional status of individuals, it is also crucial to consider the percentage of BM loss over the observation period. A loss of 5% indicates a mild and >10% a severe nutritional and health disorder [2].

Wirth et al. show that BM loss is an indicator of protein–energy malnutrition and that it increases the mortality rate of older adults in elderly homes, while BMI < 20 kg/m^2^ and weight loss > 5 kg in one year are independent and equally important risk factors for 6-month mortality. An age of 65, BMI < 20 kg/m^2^, and weight loss > 5 kg in one year are independent and equally relevant risk factors for the 6-month mortality of elderly individuals [29].

A study by Ryan et al. confirmed that older people who lost at least 5% of their total BM in a month were 4.6 times more likely to die within a year. BM is therefore a useful identifier for mortality in elderly populations. It is essential to note the data obtained from the results of the study, which show that 16 subjects (32%) were obese and their BMI > 30 [30], which approximately corresponds to the data of this research. Both malnutrition and obesity represent serious health risks and medical disorders, and great caution is needed when treating them [2].

Tracking BM loss over a long period is essential information for assessing nutritional status. Failure in the short term is primarily indicative of a disturbed balance in body fluids. In contrast, BM loss over a longer time period indicates changes in metabolism and a decrease in total tissue mass, which puts users at higher nutritional risk. Two (4%) users of this study lost TM within one month, the remaining two (4%) within two months, and nine (18%) over three months. As many as 37 users (74%) suffered no TM loss over three months (Figure S1).

When compared with the research by Ryan et al., whose data show a decrease in BM in 24/153 users (15%), this research shows a loss in only 4% (2 users) in the first month, which is much lower. Therefore, a regular application of the simple anthropometric measurement of BM can be used for detecting users at a higher risk of malnutrition, or even death. The need for further research is apparent in order to observe the role of nutrition in older populations regarding long-term care [31].

Those with reduced dietary intake in the week prior to start of investigation had statistically significantly higher BM Loss (Mann–Whitney U = 43.5; *p* < 0.001) (Figure 2). At the same time, there is a statistically significant difference in the distribution of data between users who have complete autonomy and users who have partial autonomy in feeding (*p* = 0.002). Users with full independence have been found to have higher data dispersal due to eating habits, which can be at both extremes from too much to too little food intake. In those who do not have complete independence, people who help them consume better regulate the quantities and types of foods, so this subgroup is within the boundaries of normal BMI.

Comparing the number of meals that users consume (Figure 3) shows that there is a statistically significant difference between the number of daily meals and BM (*p* = 0.041), the usual BM (*p* = 0.021), and BH (*p* = 0.037), but interestingly not with BMI-a (*p* = 0.334). BH is correlated well (r = 0.5604; *p* < 0.001) with BM, which is why there is no significant difference in BMI. Additionally, users with larger BMs and BHs typically consume more meals per day.

Constipation is common in geriatric populations due to decreased motor function of the colon, reduced fluid intake, and reduced food volume. This study shows that 4 (8%) users often had nausea, 5 (10%) diarrhea, 6 (12%) suffered from constipation, and 35 (70%) had no gastrointestinal symptoms (Figure S2). In terms of the division of users by BMI (Figure 4), most have no problems in each group. In contrast, constipation is most prevalent in the group with dangerous obesity, nausea in the group with morbid obesity, and diarrhea in the group with regular and excessive BM. In elderly homes in other countries, about 60–80% of users have constipation symptoms, which can lead to greater negative consequences and ultimately affect quality of life. Although a high prevalence of constipation in the elderly may be found, there is a lack of empirical evidence to provide interventions based on individual risk factors for constipation [31].

In six people (12%) (Figure S2) who frequently suffered from constipation, a loss of BM was observed in the past three months, citing changes in the environment and loss of taste and odor as a result of self-assessment (Figure 5). Changing the environment as a cause of BM loss is found in all those with elevated BMI and most with normal body weight. Excessive BM users are the second most common cause of BM loss, citing illness and, ultimately, the loss of a loved one. Users with normal BM, with damage caused by a change in the middle, also declared a loss of taste and smell as the main reason for their loss of BM.

The results of this study (Figure S3) show that as many as 39 (78%) users take more than three types, while only 11 (22%) take fewer than three types of medicine. This comparison (Figure 6) shows that everyone in the morbidly obese group belongs to the group that consumes more than three drugs, as do most of the other groups. Those who consume fewer than three medicines were predominantly found in the group with normal and excessive BM. These results confirm that older people suffer more from chronic diseases and take more varied drugs. Results of a study by Morais et al. in 2013, conducted on 664 older people in Europe, have shown that 50.2% of respondents take three or more medicines per day [32], which is, compared to this research, certainly fewer.

Medications can cause a disturbance in food intake by acting directly or indirectly on metabolism, and they can potentially endanger oral health. Older people commonly use anticholinergics, tricyclic antidepressants, sedatives, antihypertensives, muscle relaxants, and benzodiazepines. These drugs reduce the production of saliva, which contributes to a loss of weight and BM and also compromises nutritional status [33].

Older adults often do not consume enough fluids during the day, with the reason being that, with age, the thirst mechanism decreases and they do not feel the need for it even though it is severely deficient in the body [34]. Regarding the users of this study, 23 (46%) take up to 1 L of fluid per day, 19 (38%) < 1 L daily, and only 8 (16%) > 1 L daily (Figure S4), which in no way follows the recommended guidelines [1]. An insight into the further result obtained (Figure 7) shows that users with reduced and typical BM take < 1 L water, and those with elevated and excessive BM > 1 L water. Studies in elderly homes reveal that dehydration is common, and 20–30% of older people suffer from dehydration caused by loss of water; elderly individuals displaying dehydration-related confusion and disorientation recover either entirely or significantly once their fluid intake becomes adequate [35,36]. One prospective study found dehydration in 38.3% of elderly home users, showing that 30.5% of users are at risk of developing dehydration. Total water intake averages 1l to 1.5l, and 96% of users do not meet the estimated daily intake of fluid [37]. When compared to our research, it can be seen that the indicators of inadequate fluid intake are the same, with only 16% intaking > 1 L. In another study, fluid intake was inversely related to an increase in age, cognitive impairment, food consumption challenges, and increased dining staff, and as many as 85% of elderly home users consumed less than 1.5 L of fluid per day [38]. Other studies also confirm the low daily fluid intake of elderly home users. Reed et al. found that 51.3% of nursing home users in the United States, with an average age of 85, consumed less than 237 mL of fluid per day, with as many as 37% of those experiencing severe cognitive impairment [39]. Inadequate total fluid intake and, consequently, dehydration, prevail in the elderly population in nursing homes in all BMI categories. Nutritional interventions should be directed towards giving more attention to the type of liquid beverages, as well as diets in terms of milk, yogurt, and soup.

The reduced protein–energy status of a person causes severe health problems and affects nutritional status. This study sought to obtain as much information as possible on the intake of proteins, fats, milk, dairy products, carbohydrates, and vegetables and fruits through daily diets [8] by looking at menus (Table S1), and has made following the recommendations regarding guidelines [1]. The daily advice for protein consumption is 0.8 g/kg up to 1.5 g/kg, which can influence the prevention of muscle mass loss, i.e., sarcopenia [1]. It is evident from the survey results that 32 (64%) users consumed protein and fat several times a week, 16 (32%) every day, and only 2 (4%) once a week (Figure 8). Similar research was conducted on elderly home users who were monitored for protein status and protein intake regulated by daily diet and enteral preparations. Users had higher BMI and less pronounced symptoms of musculoskeletal pain [40].

Fat consumption in elderly homes largely depends on the type of food and how it is prepared. Research conducted in elderly homes has shown that very little is invested in the selection of quality foods with fewer saturated fatty acids, which poses a risk to the health of elderly populations. Menus in elderly homes contain very few quality sources of unsaturated fatty acids [41]; the recommended intake of total fat is 20–35% [1]. Milk and dairy products were consumed daily by 23 (46%) users, 15 (30%) several times a week, while only 3 (6%) once a week (Graph 8). Insufficient intake of milk and milk products below the recommended level causes the skeletal system to weaken. The daily calcium intake in the elderly population is 1200 mg/day, which is the same as the recommendation for vitamin D, which reduces the risk of complications of osteopenia and osteoporosis [42].

Adequate energy and protein intake could be important in the process of maintaining the health of elderly populations. The data concerning the real food intake of users in nursing homes generally are not yet sufficiently examined.

Despite nationally available UK testing guidelines for menu planning and nutrition for people in elderly homes, as many as fourteen studies show that the food offered does not meet nutritional needs. Another the survey concludes that inadequate intake of milk and dairy products is also associated with a lack of protein intake and an increased risk of malnutrition [43], which has also been shown by Van Zwien et al. (2019) [41] in their study, which states 18% of users achieved a protein intake of 68 g/day, which is less than in this current paper (46%). Croatian national guidelines point out that 50% of the elderly do not have the right amount of vitamin D due to insufficient exposure to the sun, thin skin, and reduced intake of milk, dairy products, and meat [1].

In total, 30 users (60%) in this study consume carbohydrates daily, 19 (38%) multiple times a week, and only one respondent (2%) once a week (Figure 8). The guidelines do not show traditional allowable UH intake values. Still, there is a consensus that an intake preference provided will provide 55–60% of total daily energy intake in the form of compound UHs [1]. Frequent carbohydrate intake per day significantly increases the caloric value of foods. It often causes adverse effects on the psychological function of older individuals, leading to a rapid increase in BMI and a decrease in cognitive function, especially memory [44]. A literature source shows that, in general, carbohydrate-rich foods represent the largest share in almost every single meal. The highest risk for the health of the elderly is the intake of high-calorie carbohydrates, such as refined sugars through cakes, and it has been shown that the consumption of these foods increases mortality and creates a higher incidence of heart disease and stroke in the elderly [7,41].

Consumption of vegetables by the users of this study shows that 3 (6%) consume vegetables every day, and 47 (94%) several times a week. Regarding fruit, 18 (36%) respondents consume it daily, 31 (62%) consume it several times a week, while 1 (2%) eat it once a week (Figure 8). A similar study was conducted by experts on the consumption of fruits and vegetables in a sample of 445 people over 65 years old, and the results have shown that 37% of people living in urban areas and 51% of people in rural areas do not consume the recommended five units of fruits and vegetables daily. In terms of reasoning, participants cited their inferior social status relative to the general population, decreased appetite, and taste sensation [45]. Research findings in other countries showed that few people consumed fruits or vegetables every day [46].

Exceptional care is given to nutrition in both the preparation and nutritional value of food. Food in the home is prepared in three primary meals and two entrees of the local cuisine, respecting variety and being tastefully prepared. Meals tailored to the health needs of users are also prepared: for people with diabetes, low fat and salt meals, vegetarian meals, the ability to adjust according to user preferences, mixed food served in the dining room, or tablet system in rooms (Table S1).

Users of this study self-reported changes in their diet and the elimination of or reduction in consumption of a particular group of foods. After comparing the dietary changes with the loss of BM (Figure 9), it was found that people who reduced protein and fat intake had the highest result in reducing BM. On average, the reduction in carbohydrate intake was the best. People who reduced their intake of fruit or did not reduce their consumption did not show a decrease in BM, unlike in research Morais et al., 2013 [33] showing that 53% of examinees developed nutritive risk by decreasing their consumption of fruit and vegetables (for easier chewing). It is important to draw attention to life circumstances, changes in appetite or health, general perceptions of health, intake of food and vegetables, and choice of food that is easy to chew, as all of these elements influence BMI.

Figure 10 shows how, given the consumption of a particular macronutrient source, users were divided into BMI groups. It follows that respondents who consumed vegetables daily were generally in groups with lower BMIs than others. The group with the highest incidence of morbid obesity consumed proteins and fats, carbohydrates, and vegetables several times a week.

A loss of muscle mass, resulting in a loss of strength, is also one of the essential components of the fragility of older individuals of both sexes. Moderate physical activity is recommended among older people, as it is important to strengthen muscle mass and increase strength and endurance [1]. In this study, 40 (80%) users were physically active, while only 10 (20%) were not (Figure S5). A comparison of the users of this study in different BMI groups (Figure 11) has shown that everyone with morbid obesity engages in some physical activities, thus showing that they care about their health. Additionally, most subjects with dangerous obesity and overweight play sports on their own or with help. Unfortunately, the majority of people who are a healthy weight avoid sports, which could lead them to have problems related to accumulating excess weight in later life; thus, it is recommended that the general population be motivated as much as possible to play sports. Research at the European level shows four clusters with an association between energy–protein intake and physical activity among the elderly; this is the best strategy for achieving adequate body weight [47].

The results of similar research in elderly homes link fragility with physical activity, suggesting that home users have a higher propensity for sedentary lifestyles [48]. According to the study, one hour of immobility per day confirmed the increased risk of sarcopenia by 33% [49]. A sedentary lifestyle in the elderly is associated with decreased functional ability and an increased risk of disability in daily activities [50,51]. Improper food intake can affect the functional status of older people in nursing homes. At the same time, results of a presentation of nutritional status among users in Spanish homes show an inadequate composition of the nutritional value of meals [52]. Older users who do not consume adequate energy input may not have the energy or power to access food and water on their own, request additional food or water, or feed on their own. In a sample of 98 nursing home users, it was confirmed that malnutrition predicted deterioration in functional status [53,54].

The kitchen of the elderly home in question is equipped with the latest food preparation machines and equipment, all following the HACCP system (Figure S6); this is also a legal obligation in the Republic of Croatia. It ensures the implementation of ethical manufacturing and good hygiene practices that guarantee quality technological processes and healthy and safe products. In this way, nutrient intake and its interactions with antinutrients [10], heavy metals [11], (myco) toxins [12,13,14,15], and pesticides [16] are monitored; thus, a holistic approach to the assessment of exosomes (which has advocated lately) is conducted [20,55].

This research aims to apply a form for assessing nutritional status, eating habits, and health status of users/respondents in elderly homes.

Through the process of aging, more physical and psychological changes (psychosocial and socioeconomical) are developed, which often influence nutritional needs and nutritive status. In this day and age, chronic diseases are often accompanied by therapy consumption (medicine), which can lead to an imbalance in diet and can often result in a poor nourishment status [56]. Therefore, one of the key elements regarding the care of older individuals is the regular evaluation of their nutritive status. A characteristic of the diet of this population is that their need for energy and macronutrients becomes lower, while their need for micronutrients stays the same or becomes higher than during adulthood. Therefore, the organizing of meals is exceptionally important [57].

The data of a large study encompassing 60 hospitals in the evaluation of the nutritive status of patients through the use of different forms/tools, using SGA, showed that 63.3% of examinees were malnourished, while the application of NRS showed that 90% of hospitalized elderly people were malnourished [58].

Therefore, another goal of this paper is to obtain insight into the foodomics approach, which plays a key role in personalized diets and the optimization of diets for older individuals.

This paper sought to obtain insights not only regarding the nutritional value of prepared meals, but also in terms of the intake and interaction between individual portions of food by assessing exposures, as well as through the application of the HACCP system to ensure that meals were prepared correctly in these institutions.

## 5. Conclusions

In terms of normal BM, 14 (28%) users have a BMI ranging from 20 kg/m^2^ to 25 kg/m^2^. Eighteen users (36%) are overweight, which exceeds the BMI limits of 25 kg/m^2^ to 30 kg/m^2^. This means that 13 (26%) users are in Grade I obesity, and two (4%) are in Grade II; meanwhile, one user is in Grade III, which signifies morbid obesity with a BMI > 40 kg/m^2^

Users with reduced dietary intake in the week prior to investigation had statistically significantly higher BM loss (Mann–Whitney U = 43.5; *p* < 0.001).

At the same time, there is a statistically significant difference in the distribution of data between users who have complete autonomy and users who have partial autonomy in feeding (*p* = 0.002); users with full independence have been found to have higher data dispersal due to their eating habits.

Comparing the number of meals that users consume shows that there is a statistically significant difference between the number of daily meals and BM (*p* = 0.041), the usual BM (*p* = 0.021), and BH (*p* = 0.037), but interestingly not with BMI- a (*p* = 0.334). BH is correlated well (r = 0.5604; *p* < 0.001) with BM, which is why there is no such significant difference in BMI. Additionally, users of larger BMs and BHs typically consume more meals per day.

This research highlights the importance of checking and continuously monitoring the nutritional status of those in elderly homes to prevent nutritional risk. It is also essential to ensure the preparation of a nutritionally valuable healthy meal by applying and implementing an HACCP system in addition to technologically traditional forms of food processing that seek to satisfy a holistic approach to assessing exposures. Furthermore, the use of an individual and comprehensive approach in elderly homes has to be emphasized in order to ensure adequate nutritional intake of food and liquids, to improve the health status of users, and to advance the quality of life of elderly people.

All of these elements are connected in foodomics as a tool for the development of healthy food that is adapted to individual health challenges and the prevention of illnesses connected to food in order to improve the increasingly longer lifetime of humans.

Diet problems, which often result in malnutrition, are generally spread among elderly people in nursing homes. The prevalence rates differ depending on the parameters and limit values used for evaluating nutrition, as well as on the examined population. Future studies should carefully characterize their participants and use standardized tools for evaluating nutrition in order to achieve a better comparison of their results.

## Supplementary Materials

The following are available online at www.mdpi.com/article/10.3390/foods10102391/s1, Supplementary file 1—Content: Questionnaire S1: An individualized questionnaire prepared according to the guidelines of standardized methods for assessing the nutritional status of the elderly for research purposes, Figure S1: Distribution of users according to weight loss over a period of 1 month, 2 months and 3 months in absolute and relative terms, Figure S2: Review of the user’s response to a question about the presence of gastrointestinal symptoms in absolute and relative terms, Figure S3: Review of user’s response to a question about taking medication and the effect of therapy on weight loss in absolute and relative terms, Figure S4: Distribution of users according to fluid intake daily in absolute and relative terms, Figure S5: Review user’s response to a question: Are you physically active? in absolute and relative terms, Figure S6: certification of HACCP system certification. Table S1: The menu of nursing home used for research.
