# Supplementary material for: Determination of the Personal Nutritional Status of Elderly Populations Based on Basic Foodomics Elements
Source: Foods. 2021 Oct 9;10(10):2391. doi: 10.3390/foods10102391 (PMC8535163; doi:10.3390/foods10102391)
Supplement: Supplementary file 1 [file foods-10-02391-s001.zip › foods-1367908-supplementary.pdf]

Questionere S1

An individualized questionnaire prepared according to the guidelines of standardized methods for assessing the nutritional status of the elderly for research purposes

1. Gender of respondents:
2. Age of respondents:
3. Body height of respondents:
4. Bodyweight of respondents:
5. BMI (Body mass index) of respondents:
6. Your weight loss occurred in the past:

a) 1 month
b) 2 months
c) 3 months
d) No loss of body weight

7. Weight lost in the past3 months:

a) < 5 kg
b) 5 kg
c) more than 5 kg
d) 0 kg

8. Weight loss in your diet has been due to:

a) illness
b) change of residence (environment)
c) loss of a loved person
d) independent life
e) loss of taste and smell
f) nothing

9. Do you take more than 3 types of medication?

a) yes
b) no

10. Does taking medication/therapy affect your weight loss?

a) yes
b) no

11. Are you physically active (eg walking…)?
a) yes
b) no
12. The number of daily meals:
a) 2
b) 3
c) more than 3
13. How much water/liquid do you drink daily?
a) less than 1-liter
b) 1-liter
c) more than 1 liter
14. How often do you consume protein and fat (meat, fish, legumes, fats)?
a) every day
b) several times a week
c) once a week
15. How often do you consume milk and dairy products?:
a) every day
b) several times a week
c) once a week
d) I do not consume
16. How often do you consume carbohydrates (bread, pasta, cakes)?:
a) every day
b) several times a week
c) once a week
17. How often do you consume vegetables?:
a) every day
b) several times a week
c) once a week
18. How often do you consume fruits?
a) every day
b)several times a week
c) once a week

19. Metabolic needs due to disease:
a) Do you have an increased need to eat a certain food?
b) is there a prescribed diet due to the disease?
c) no metabolic needs
20. Have you recently reduced in your diet the intake of:
a) protein and fat
b) milk, dairy products
c) carbohydrates
d) vegetables
e) fruits
f) nothing
21. How long does such a change in your diet last):
a) 3 months
b) 6 months
c) 12 months
d) no change
22. Has your current state of health affected the change in your diet recently?
a) yes
b) no
c) I can't estimate, I don't know
23. Do you have problems with:
a) nausea
b) vomiting
c) diarrhoea
d) digestive tract constipation
e) nothing
24. Level of independence in consuming meals:
a) complete independence
b) partial independence

Figure S1

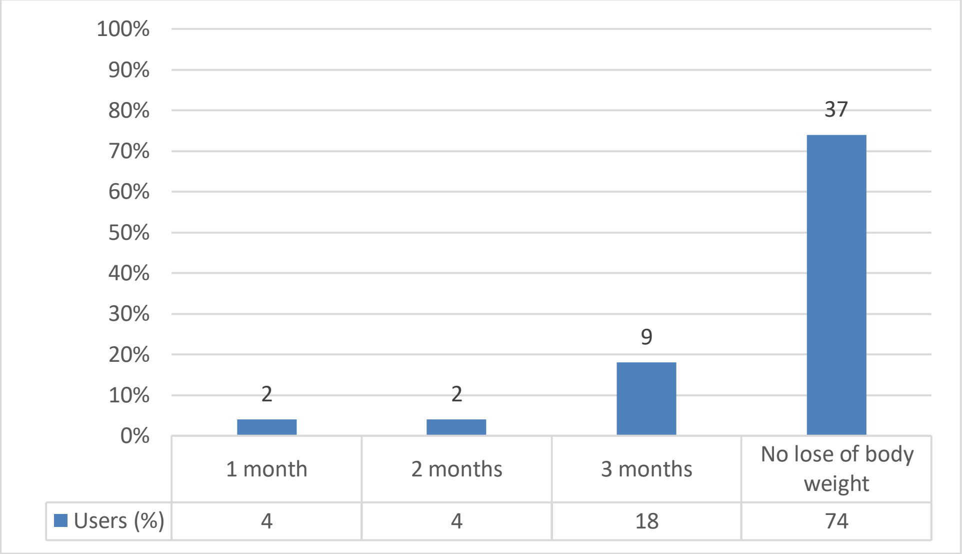

Distribution of users according to weight loss over a period of 1 month, 2 months and 3 months in absolute and relative terms.

Figure S2

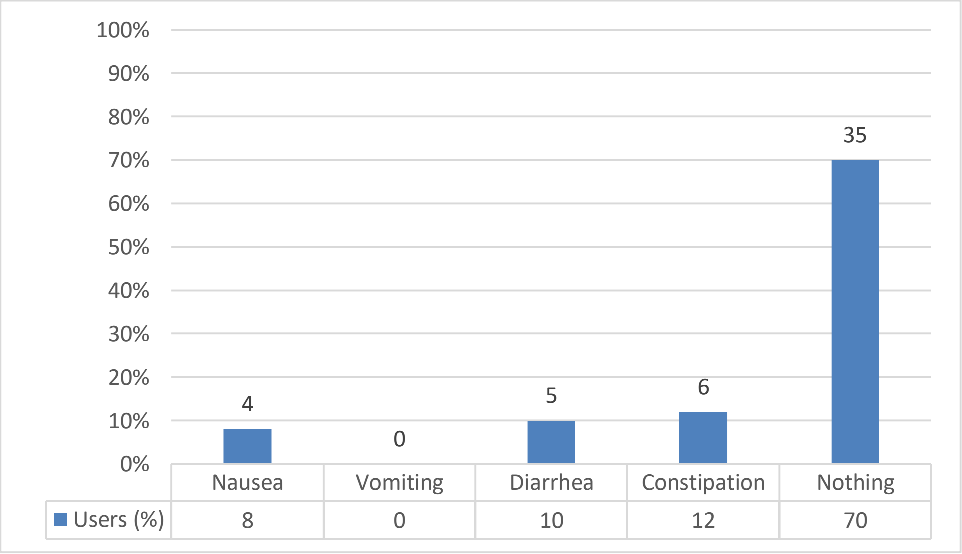

Rewiev of the user's response to a question about the presence of gastrointestinal symptoms in absolute and relative terms

Figure S3

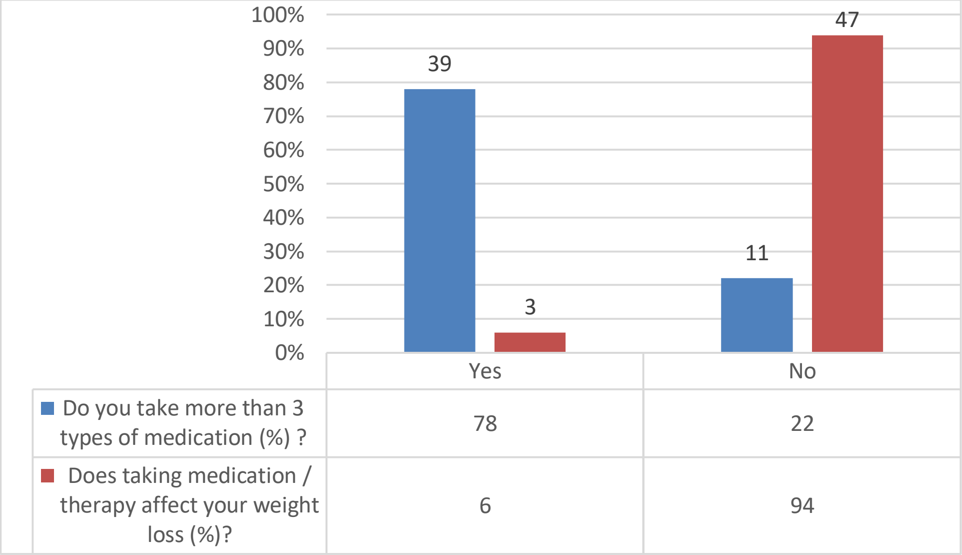

Rewiev of user's response to a question about taking medication and the effect of therapy on weight loss in absolute and relative terms

Figure S4

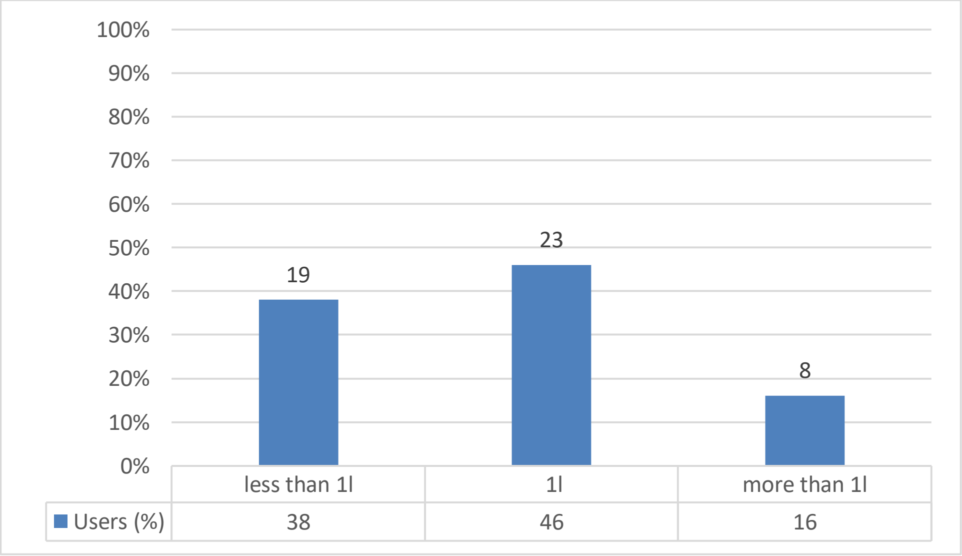

Distribution of users according to fluid intake daily in absolute and relative terms

Figure S5

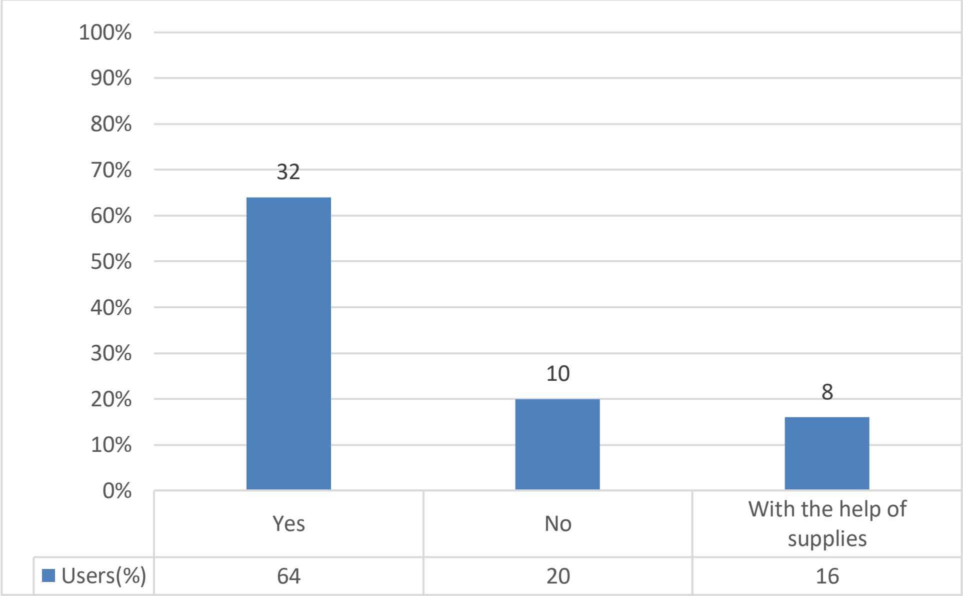

Rewiev user's response to a question: Are you physically active? in absolute and relative terms

Table S1

The menu of nursing home used for research

DAY	MELAS	STANDARD DIET	THE GALLBLADDER DIET	DIABETIC DIET	MUSHY DIET
MON	BREAKFAST	Cocoa, mixed tea, melted cheese	Cocoa, mixed tea, melted cheese	Cocoa, mixed tea, melted cheese	Cocoa
	LUNCH	Paste, bean, sausage	Cauliflower stew, potato, sausage	Paste, bean, sausage	Cauliflower stew, potato, sausage
	DINNER	Baked dough, mixed tea	Baked dough, mixed tea	Baked dough, mixed tea	Baked dough, milk
	SNACK	/	/	Yogurt 0,9%, Kefir	/
TUE	BREAKFAST	White coffee, mixed tea, pašteta	White coffee, mixed tea, butter	White coffee, mixed tea, butter	Cornflakes
	LUNCH	Beef soup, stewed cabbage, boiled pork, mashed potatoes, donut	Beef soup, stewed cabbage, boiled pork, mashed potatoes, donut	Beef soup, stewed cabbage, boiled pork, mashed potatoes, donut	Beef soup, stewed cabbage, boiled pork, mashed potatoes, donut
	DINNER	Rice on milk, scrambled egg soup, cookies petit	Rice on milk, cookies petit	Rice on milk, cookies petit	Rice on milk, cookies petit
	SNACK	/	/	Yogurt 0,9%, sour milk	/
WEN	BREAKFAST	Cocoa, mixed tea, butter and marmalade	Cocoa, mixed tea, butter and marmalade	Cocoa, mixed tea, butter and marmalade	Cocoa
	LUNCH	Vegetable stew, poppy pads	Vegetable stew, apple pads	Vegetable stew, apple pads	Vegetable stew
	DINNER	Dumplings with cheese, mixed tea	Dumplings with cheese, mixed tea	Dumplings with cheese, mixed tea	Dumplings with cheese, milk
	SNACK	/	/	Kefir, melted cheese	/
THU	BREAKFAST	White coffee, mixed tea, LinoLada	White coffee, mixed tea, butter	White coffee, mixed tea, butter	White cofee
	LUNCH	Beef soup, lasagne with beef, green salad	Beef soup, lasagne with beef, green salad	Beef soup, lasagne with beef, green salad	Beef soup, lasagne with beef, green salad, stewed cabbage
	DINNER	Millet porridge soup, an apple	Millet porridge soup, an apple	Millet porridge soup, an apple	Millet porridge soup, an apple
	SNACK	/	/	Sour milk, cookies petit	/
FRI	BREAKFAST	Cocoa, mixed tea, fresh cheese	Cocoa, mixed tea, fresh cheese	Cocoa, mixed tea, fresh cheese	Cocoa
	LUNCH	Vegetable soup,breaded fish steak, baked cheese, potato salad	Vegetable soup,breaded fish steak, baked cheese, potato salad	Vegetable soup,breaded fish steak, baked cheese, potato salad	Vegetable soup,breaded fish steak, baked cheese, salt potato
	DINNER	White cofee, yogurt 0,9%, corn mixed spirits	White cofee, yogurt 0,9%, corn mixed spirits	White cofee, yogurt 0,9%, corn mixed spirits	White cofee, corn mixed spirits
	SNACK	/	/	Yogurt 0,9%, an orange	/
SAT	BREAKFAST	White cofee, mixed tea, butter and honey	White cofee, mixed tea, butter and honey	White cofee, mixed tea, butter and honey	Cornflakes
	LUNCH	Beef soup, cabbage stew, mashed potato	Beef soup, cabbage stew, mashed potato	Beef soup, cabbage stew, mashed potato	Beef soup, cabbage stew, mashed potato
	DINNER	Chicken soup, an orange	Chicken soup, an orange	Chicken soup, an orange	Chicken soup, an orange
	SNACK	/	/	Sour milk, kefir	/
SUN	BREAKFAST	White cofee, mixed tea, ham	White cofee, mixed tea, ham	White cofee, mixed tea, ham	White cofee
	LUNCH	Beef soup, roast chicken, grinders, green salat, puding cake	Beef soup, roast chicken, grinders, green salat, puding cake	Beef soup, roast chicken, grinders, green salat, puding cake	Beef soup, roast chicken, grinders, stewed cabbage
	DINNER	Apple pie, mixed tea, Yogurt 2,8%	Apple pie, mixed tea, Yogurt 2,8%	Apple pie, mixed tea, Yogurt 2,8%	Cokolino, mixed tea, Yogurt 2,8%
	SNACK	/	/	Yogurt 0,9%, sour milk	/

Figure S6

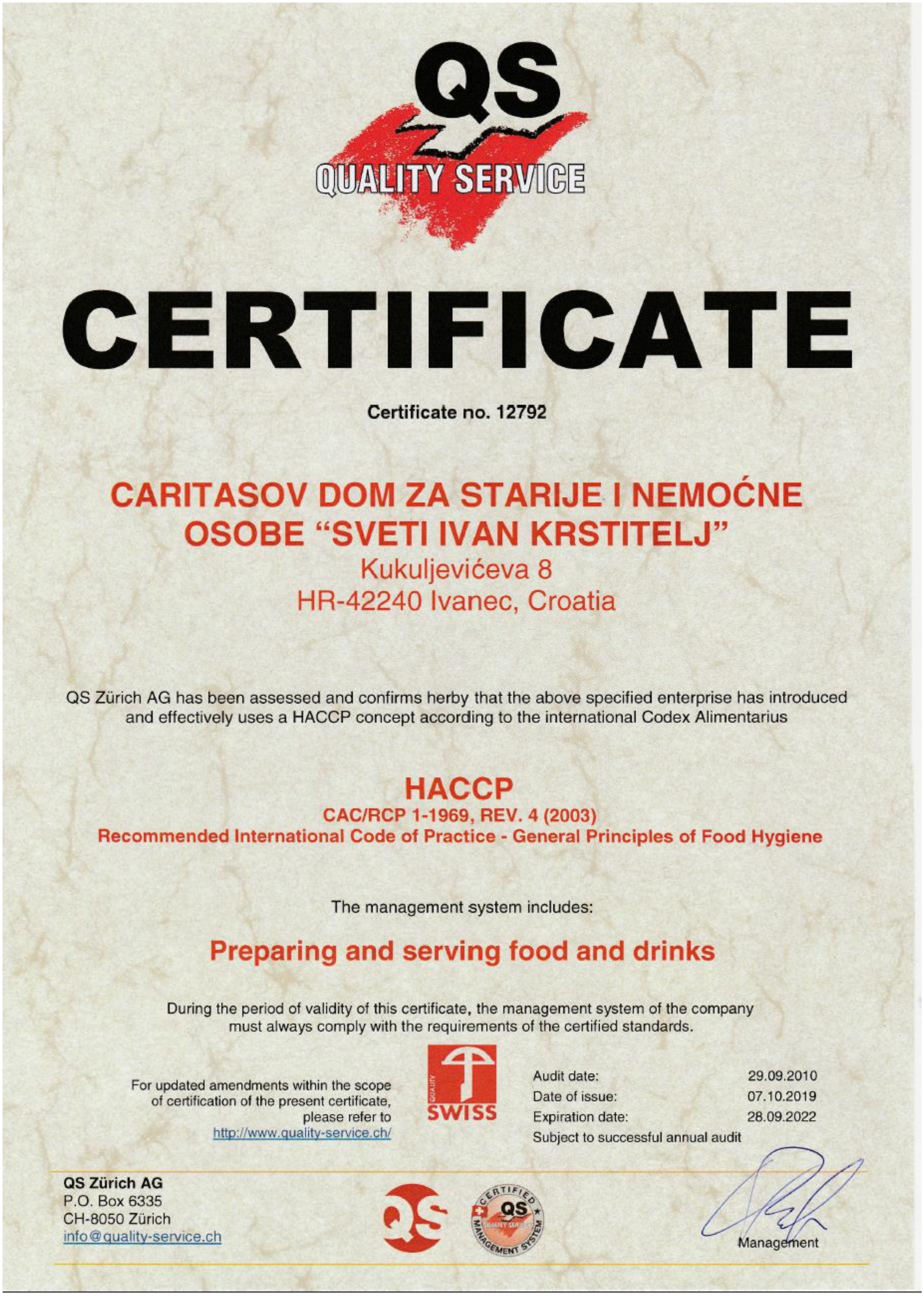

QS
QUALITY SERVICE

CERTIFICATE

Certificate no. 12792

CARITASOV DOM ZA STARIJE I NEMOĆNE OSOBE “SVETI IVAN KRSTITELJ”

Kukuljevićeva 8
HR-42240 Ivanec, Croatia

QS Zürich AG has been assessed and confirms herby that the above specified enterprise has introduced and effectively uses a HACCP concept according to the international Codex Alimentarius

HACCP

CAC/RCP 1-1969, REV. 4 (2003)
Recommended International Code of Practice - General Principles of Food Hygiene

The management system includes:

Preparing and serving food and drinks

During the period of validity of this certificate, the management system of the company must always comply with the requirements of the certified standards.

For updated amendments within the scope of certification of the present certificate, please refer to http://www.quality-service.ch/

QUALITY SWISS

Audit date:	29.09.2010
Date of issue:	07.10.2019
Expiration date:	28.09.2022

Subject to successful annual audit

QS Zürich AG
P.O. Box 6335
CH-8050 Zürich
info@quality-service.ch

QS CERTIFIED QS QUALITY SERVICE MANAGEMENT SYSTEM

Management
